# Intermittent Resistance Training at Moderate Altitude: Effects on the Force-Velocity Relationship, Isometric Strength and Muscle Architecture

**DOI:** 10.3389/fphys.2018.00594

**Published:** 2018-05-24

**Authors:** Antonio J. Morales-Artacho, Paulino Padial, Amador García-Ramos, Alejandro Pérez-Castilla, Javier Argüelles-Cienfuegos, Blanca De la Fuente, Belén Feriche

**Affiliations:** ^1^Department of Physical Education and Sport, Faculty of Sport Sciences, University of Granada, Granada, Spain; ^2^High Performance Center of Sierra Nevada, Spanish Sport Council, Granada, Spain

**Keywords:** hypoxia, strength training, muscle, power training, muscle architecture

## Abstract

Intermittent hypoxic resistance training (IHRT) may help to maximize the adaptations following resistance training, although conflicting evidence is available. The aim of this study was to explore the influence of moderate altitude on the functional, neural and muscle architecture responses of the quadriceps muscles following a power-oriented IHRT intervention. Twenty-four active males completed two 4-week consecutive training blocks comprising general strengthening exercises (weeks 1–4) and power-oriented resistance training (weeks 5–8). Training sessions were conducted twice a week at moderate altitude (2320 m; IHRT, *n* = 13) or normoxia (690 m; NT, *n* = 11). Training intensity during the second training block was set to the individual load corresponding to a barbell mean propulsive velocity of 1 m·s^−1^. Pre-post assessments, performed under normoxic conditions, comprised quadriceps muscle architecture (thickness, pennation angle and fascicle length), isometric maximal (MVF) and explosive strength, and voluntary muscle activation. Dynamic strength performance was assessed through the force-velocity relationship (F_0_, V_0_, P_0_) and a repeated CMJ test (CMJ_15MP_). Region-specific muscle thickness changes were observed in both training groups (*p* < 0.001, $\eta_{\text{G}}^{2}$ = 0.02). A small opposite trend in pennation angle changes was observed (ES [90% CI]: −0.33 [−0.65, −0.01] *vs*. 0.11 [−0.44, 0.6], in the IHRT and NT group, respectively; *p* = 0.094, $\eta_{\text{G}}^{2}$ = 0.02). Both training groups showed similar improvements in MVF (ES: 0.38 [0.20, 0.56] *vs*. 0.55 [0.29, 0.80], in the IHRT and NT group, respectively; *p* = 0.645, $\eta_{\text{G}}^{2}$ < 0.01), *F*_0_ (ES: 0.41 [−0.03, 0.85] *vs*. 0.52 [0.04, 0.99], in the IHRT and NT group, respectively; *p* = 0.569, $\eta_{\text{G}}^{2}$ < 0.01) and *P*_0_ (ES: 0.53 [0.07, 0.98] vs. 0.19 [−0.06, 0.44], in the IHRT and NT group, respectively; *p* = 0.320, $\eta_{\text{G}}^{2}$ < 0.01). No meaningful changes in explosive strength performance were observed. In conclusion, contrary to earlier adverse associations between altitude and resistance-training muscle adaptations, similar anatomical and functional muscle strength responses can be achieved in both environmental conditions. The observed region-specific muscle thickness changes may encourage further research on the potential influence of IHRT on muscle morphological changes.

## Introduction

Since the well-established “live high, train low” altitude training studies developed in the nineties (Levine and Stray-Gundersen, 1997), the use of hypoxia in sport has evolved from a mainly hematological aim to a broader scope embracing also neuromuscular aspects (Scott et al., 2014b; Brocherie et al., 2015). Early hypotheses, presenting altitude as a harmful environment for skeletal muscle development (Ferretti et al., 1990; Narici and Kayser, 1995), have been progressively challenged by the idea of using hypoxia to maximize muscle strength responses (Deldicque and Francaux, 2013; Millet et al., 2013; Feriche et al., 2017). Accordingly, it has been suggested that combining systemic hypoxia during training, and normoxia during recovery (i.e., intermittent hypoxic resistance training, IHRT), could confer an advantageous stimulus to maximize the muscle adaptations following resistance training (Scott et al., 2014a). Moreover, while two to 4-week altitude training camps are usually carried out in elite sport, an IHRT strategy could be used to target other training goals requiring longer adaptation periods (i.e., strength training related). Notwithstanding, studies exploring the influence of IHRT on muscle functional and physiological adaptations remain inconclusive and limited to normobaric hypoxic simulated conditions.

Despite greater acute hormonal and metabolic stress responses following resistance exercise under hypoxic conditions (Kon et al., 2010; Kurobe et al., 2015), there are studies reporting both significant (Nishimura et al., 2010; Manimmanakorn et al., 2013b; Kurobe et al., 2015) and no meaningful effects (Friedmann et al., 2003; Ho et al., 2014; Kon et al., 2014) of IHRT on the muscle cross-sectional area (CSA). Although differences in crucial resistance-training methodological aspects, such as the resting time between training sets, may influence the lack of agreement between IHRT studies reporting changes in muscle CSA (Scott et al., 2017a,b), other constraints in the current literature also limit the translation of these findings to the field. Resistance training practices aiming to enhance athletic performance often comprise maximal intended explosive efforts leading to both training-specific structural (Blazevich et al., 2003) and neural adaptations (Buckthorpe et al., 2015). Most of the IHRT interventions, however, have been focused on hypertrophy-oriented single-joint exercises (Nishimura et al., 2010; Manimmanakorn et al., 2013b) without considering the relevance of performing maximal intended efforts to enhance explosive muscle performance. To our knowledge, no previous studies have explored the potential effects of IHRT on other functionally relevant structural (i.e., muscle architecture) and neural (i.e., rate of muscle activation) factors.

Acute exposures to moderate hypobaric (i.e., terrestrial moderate hypoxia), but not normobaric hypoxia (Feriche et al., 2014; Scott et al., 2014a, 2017a,b), have been described to enhance muscle performance during the bench-press (Feriche et al., 2014) and squat jump exercises (García-Ramos et al., 2016). Although the underlying mechanisms are unclear, these findings suggest that IHRT under hypobaric conditions could lead to positive neuromuscular adaptations. Despite the limited evidence, hypoxia-induced changes in motor unit recruitment patterns could affect the neuromuscular responses following resistance training (Manimmanakorn et al., 2013b). Inconsistent evidence, however, comes from normobaric IHRT studies showing greater (Manimmanakorn et al., 2013b; Inness et al., 2016) and similar adaptations in maximal strength (Friedmann et al., 2003; Nishimura et al., 2010; Ho et al., 2014; Kon et al., 2014) compared to normoxia. The fact that very different maximal strength testing methodologies have been previously used [i.e., isometric (Manimmanakorn et al., 2013b; Ho et al., 2014), isokinetic (Friedmann et al., 2003; Ho et al., 2014) and direct (Kon et al., 2014; Inness et al., 2016), or indirect (Nishimura et al., 2010) maximal dynamic strength)] could partially explain these inconsistencies. Moreover, despite the significance of assessing the neuromuscular maximal mechanical capabilities, comprising both maximal and explosive strength performance (Samozino et al., 2012; Maffiuletti et al., 2016), no previous IHRT studies have considered it. A close examination of the force-velocity (FV) relationship, comprising performance assessment under multiple loading conditions (Jaric, 2015), as well as specific maximal and explosive isometric strength assessments (Maffiuletti et al., 2016) may help to reach a better understanding of the effects of IHRT on muscle strength performance.

Accordingly, the purpose of the present study was to explore the effects of a power-oriented IHRT intervention performed at moderate altitude on the functional (i.e., maximal and explosive measures of muscle strength), neuromuscular (i.e., voluntary EMG activation) and morphological (i.e., muscle architecture) responses. It was hypothesized that resistance training at moderate altitude would lead to greater adaptations in muscle strength, neuromuscular and morphological responses.

## Materials and methods

### Participants

Twenty-seven physically active male Sport Science students (age: 22.5 ± 3.4 years, height: 177.0 ± 7.1 cm, body mass: 76.1 ± 8.5 kg) volunteered to participate in this study. All participants were informed regarding the nature, aims and risks associated with the experimental procedures and provided informed consent. Participants were familiarized with the resistance training exercises, although none of them was involved in systematic resistance training programme at the beginning of the study. None of them reported any physical limitations, health problems or musculoskeletal injuries that could compromise testing. Due to personal reasons, three participants withdrew from the study and 24 completed the training intervention (Hypoxia, *n* = 13 and Normoxia, *n* = 11). All participants were lowlanders and had not previously carried out any exercise training at altitude. All subjects gave written informed consent in accordance with the Declaration of Helsinki. The protocol was approved by the Granada University Ethic Committee.

### Experimental design

A longitudinal repeated measures design was used to evaluate the functional and physiological muscle adaptations following resistance training at intermittent moderate altitude or normoxic conditions. One week before official testing, two familiarization sessions comprising loaded CMJ were carried out. Afterwards, participants attended the laboratory for three official testing sessions before (2–4 days pre-training) and after (2–4 days post-training) the 8-week training intervention. On days 1 and 2, unilateral knee extension isometric force, voluntary muscle activation (surface electromyography, EMG) and ultrasound-based muscle architecture repeated assessments were performed. Specific separate testing protocols were performed to measure maximal and explosive voluntary isometric force (Maffiuletti et al., 2016). In order to assess the between-day reliability of isometric and ultrasound measurements, assessments carried out on day 1 were repeated on a separate day. On the third visit to the laboratory (48 h later), participants first carried out a progressive loading FV test during the CMJ exercise and then performed a repeated jumping test consisting of 15 continuous CMJ repetitions at maximum intended velocity. All laboratory assessments were performed under normoxic conditions (i.e., 690m) and participants were asked to refrain from physical efforts, alcohol intake and maintain their sleep and diet habits 48 h before assessments. The training intervention comprised two continuous 4-week training periods (16 training sessions in total) of resistance training under normoxic environmental conditions (690 m; NT group) or intermittent terrestrial moderate altitude (at the High Performance Center of Sierra Nevada, 2,320m; IHRT group). Participants in the IHRT group traveled 32Km by car to altitude to complete each training session and returned to normoxic conditions. Arrivals and departures from altitude training took place ~20 min before and after the corresponding training session. The hypoxic environmental conditions during IHRT were ensured by assessing the arterial oxygen saturation (SaO_2_) before each training session. Moreover, all participants were asked to avoid any lower-body resistance training activity out of the study.

### Training intervention

Training sessions were performed twice weekly, with at least a 48 h rest between them. The training intervention consisted of two-consecutive 4-week resistance training periods. In order to optimize the power training adaptations (Cormie et al., 2010b), the first 4 weeks of training comprised bodyweight strength exercises, loaded squats and deadlifts and were designed to enhance general strength levels and CMJ jumping technique. During the back squat and deadlift exercises, the training load was individually set to ~12 RM. Two approximation sets (i.e., at 50 and 80% of their perceived 12 RM load) during the specific warm-up for each exercise were used to determine the individual training load for each session. This exercise program design was aiming to maximize muscle adaptations following lower-body explosive training (Zamparo et al., 2002; Haff and Nimphius, 2012). During the second 4-week training period, sessions were designed to improve both maximal power development and repeated jumping performance. A detailed description of the training program (i.e., exercises, sets, repetitions, rest periods, etc.) is shown in Table 1. All loaded CMJ were performed in a Smith Machine (Technogym, Gambettola, Italy) and the external load used was selected for every participant as the load associated with a barbell mean propulsive velocity of 1 m·s^−1^ (see below training load estimation procedure). Additionally, 5 min before the warm-up, the SaO_2_ levels were measured using a pulse oximeter (Onyx Vantage 9590, Nonin, Plymouth, MN, USA). The IHRT group displayed a mean SaO_2_ value of 93.6 ± 2.1%.

**Table 1:** Training program description during the 8 weeks of training.

**Weeks 1–4**								**Weeks 5–8**			
**Sessions 1 and 3**		**Sessions 2 and 4**		**Sessions 5 and 7**		**Sessions 6 and 8**		**Sessions 9, 11, 13, and 15**		**Sessions 10, 12, 14 and 16**	
**Exercise**	**Sets × reps (rest)**	**Exercise**	**Sets × reps (rest)**	**Exercise**	**Sets × reps (rest)**	**Exercise**	**Sets × reps (rest)**	**Exercise**	**Sets × reps (rest)**	**Exercise**	**Sets × reps (rest)**
Jumping Jacks	6 × 50 (1 min)	Jumping rope	6 × 50 (1 min)	Jumping Jacks	3 × 50 (30 s)	Jumping rope	3 × 50 (30 s)	Jumping Jacks	3 × 20 (30 s)	Jumping Jacks	3 × 20 (30 s)
Air squat	6 × 25 (1 min)	Step-up^c^	6 × 20 (1 min)	Box jumps^c^	5 × 10 (3 min)	Drop jumps^c^	5 × 10 (3 min)	Drop jumps^c^	4 × 10 (4 min)	Loaded CMJ^e^	5 × 6 (5 min)
Back Squat^a^	3 × 10 (2 min)	Back Squat^a^	3 × 10 (2 min)	CMJ^d^	5 × 10 (3 min)	Free barbell CMJ	5 × 10 (3 min)	Loaded CMJ	5 × 6 (5 min)	Loaded CMJ^e^	5 × 15 (3 min)
Romanian Deadlift^a^	3 × 10 (2 min)	Single-leg Deadlift^b^	3 × 10 (1 min)	Romanian deadlift^a^	3 × 10 (2 min)	Single-leg deadlift^b^	3 × 10 (1 min)	Sit-up	3 × 25 (30 s)	Back extension	3 × 25 (30 s)
Sit-up	5 × 20 (30 s)	Deadlift^a^	3 × 10 (2 min)	Sit-up	5 × 20 (30 s)	Good-morning^b^	3 × 10 (1 min)				

All training sessions were preceded by a standardized warm-up protocol consisting of 5 min jogging and mobility exercises.

a Training load (kg) approximated to 12 repetitions maximum and determined through 2 approximation sets

b Single-leg deadlift load (kg) was 20 kg for all participants.

c Step and box height was 40 cm.

d CMJ was performed with a free barbell. External load (kg) was 25% of each participant's body weight.

e The external load (kg) was interpolated from the corresponding load-velocity relationship. These were assessed prior to the loaded CMJ exercise in sessions 9, 11, 13 and 15.

#### Training load estimation

During the second 4-week period, the external load used during all loaded CMJ was estimated from the load-velocity relationship for each participant on a weekly basis. Precisely, during the 1st weekly training session, the load-velocity relationship was constructed by performing two CMJ attempts at three different absolute loading conditions (i.e., 20, 40, and 60 kg). A linear regression model was fitted and used to estimate the external load corresponding to a barbell mean propulsive velocity of 1 m·s^−1^. This load is known to be equivalent to ~50–55% 1RM during the CMJ exercise (Pérez-Castilla et al., 2017). This procedure ensured that training loads were adjusted in both experimental conditions, and thus participants trained at the same relative intensity, for the same prescribed training volume.

### Pre- and post-training assessments

#### Knee extension isometric maximal and explosive strength

**Figure 1:**
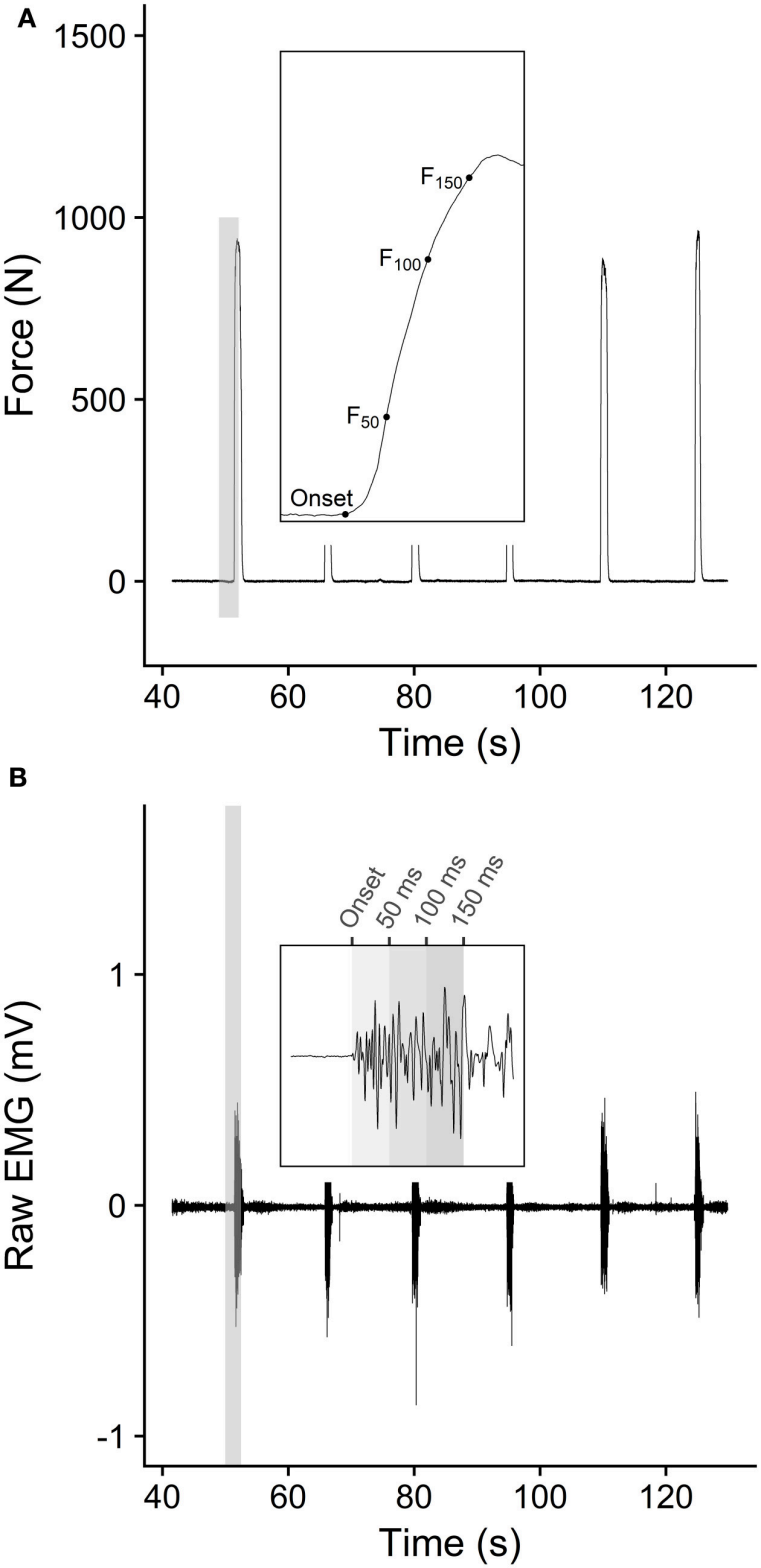
**(A)** Typical example of force trace during an isometric explosive strength assessment. Each force onset was automatically detected as the last zero-crossing point on the first derivative of the filtered signal force (de Ruiter et al., 2007), and force values at 50, 100, and 150 ms were compared. **(B)** Typical example of EMG recording during the isometric contractions. Signal onset was manually identified and the root means square (RMS) was averaged across 0–50 ms, 50–100 ms, and 100–150 ms time periods after onset.

Maximal and explosive voluntary strength assessments were performed on a custom-made isometric rigid dynamometer (Maffiuletti et al., 2016) with knee and hip angles of 110° and 130° (180° = full extension), respectively. Pelvis and shoulders were firmly secured to the chair and a rigid strap was attached proximally to the ankle (2 cm above the lateral malleolus) in series with a calibrated low-noise strain gauge (Force Logic, Swallowfield, UK). The force signal from the strain gauge was amplified (×370) and digitized at 1 kHz using a 16-bit analog to digital converter (DT 9804; Data Translation, Marlboro, Massachusetts, USA). Following a standardized warm-up (three sustained contractions for 3–4 s at 20, 40, 60, and 80% of maximal perceived exertion), participants performed three maximal voluntary contractions (MVC) extending their knee “as hard as possible” for 3–5 s. Resting periods between efforts were set to 1 min. Thereafter, participants performed 10 explosive voluntary contractions interspersed by 30 s resting periods. They were instructed to push “fast, then strong” for ~1 second and to avoid any countermovement or pre-tension prior to the force onset (Maffiuletti et al., 2016). Force signals recorded from the strain gauge were filtered with a 4th order 150-Hz low pass Butterworth filter and were corrected for the influence of gravity during offline analysis. The highest instantaneous peak force achieved during the MVCs was defined as maximal voluntary force (MVF). Force signals recorded during the explosive voluntary contractions were first analyzed to determine onset and to discard any attempt with pre-tension or countermovement. Force onsets were automatically identified and visually confirmed (Tillin et al., 2013), as the last zero-crossing point on the first derivative of the filtered signal force (de Ruiter et al., 2007). To discard any contraction with pre-tension or countermovement, 100 ms baseline force signals prior to force onset were fitted with a least-squares linear regression, and absolute slope values >1.5 N·m·s^−1^ were set as a criterion for contraction omission due to potential effects of co-contraction or countermovement on explosive performance (de Ruiter et al., 2007). Explosive contractions reaching peak force < 75% MVT were also discarded. From the remaining explosive voluntary contractions, the three attempts with the highest force at 100 ms were further analyzed and eventually averaged across. For each contraction, force at 50 ms (F_50_), 100 ms (F_100_), and 150 ms (F_150_) after the force onset were used for further analyses (Figure 1A).

#### Surface electromyography (EMG)

During all isometric force assessments, EMG activity was recorded wirelessly from the *vastus medialis* (VM), *vastus lateralis* (VL), and *rectus femoris* (RF) muscles (Delsys, Boston, Massachusetts, USA). Raw EMG signals were amplified (× 1,000) and sampled at 2 kHz. After skin preparation (shaving, light abrasion and cleaning with alcohol), EMG surface electrodes (Trigno Standard Sensor; Delsys Boston, Massachusetts, USA) were placed according to the surface EMG for non-invasive assessment of muscle recommendations (Hermens et al., 2000). EMG signals were first filtered with a 4th order band-pass Butterworth filter (6–450 Hz). The EMG signal during MVCs was assessed with a 500 ms root mean square (RMS) epoch, 250 ms either side of the peak EMG (Buckthorpe et al., 2012), averaged across muscles and taken as maximal EMG activation during MVC (EMG_MVC_). During explosive contractions, EMG onset was manually identified as the first muscle to be activated. Specifically, raw EMG signals were graphically displayed with systematic x and y-axis (i.e., 300 ms and ± 0.05 mV, respectively) before manually selecting the last point at which the signal deflected away from baseline (Balshaw et al., 2016). Thereafter, RMS EMG was averaged over three periods from the EMG onset: 0–50 ms, 50–100 ms, and 100–150 ms (EMG_0−50_, EMG_50−100_, EMG_100−150_, respectively), normalized to EMG_MVF_ and averaged across muscles (Figure 1B).

#### Muscle architecture

**Figure 2:**
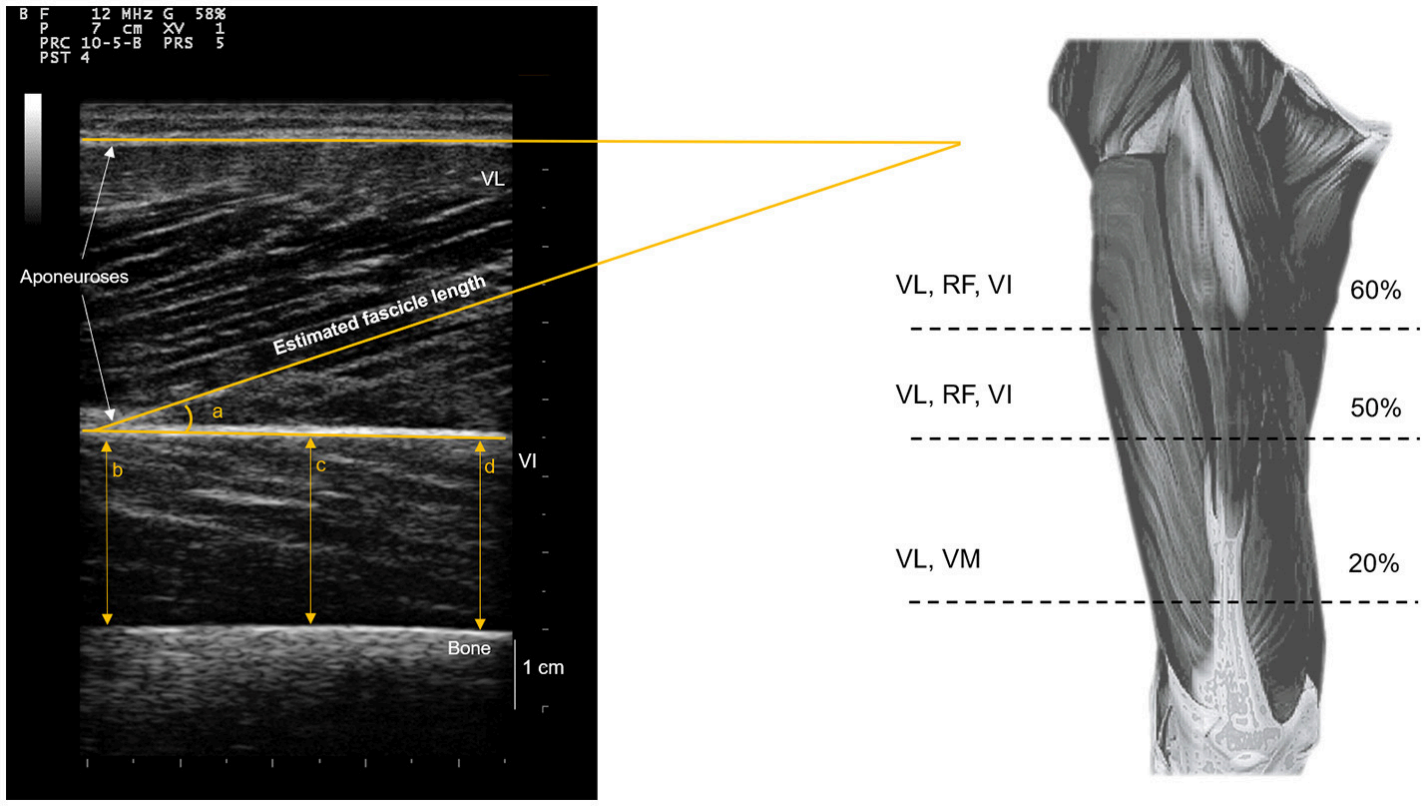
Muscle architecture assessments. Muscle thickness measurements on vastus lateralis (VL), rectus femoris (RF), vastus medialis (VM), and vastus intermedius (VI) were performed at each corresponding measurement location (% thigh length). Muscle thickness VI measurements were performed in the anterior portion, in the same location as RF measurements. Pennation angles and linear estimations of fascicle lengths were averaged across the three VL muscle measurements.

Figure 2 shows an overview of the muscle architecture measurements carried out. For each measurement site, muscle thickness was measured as the perpendicular distance between superficial and deep aponeuroses, averaged at three evenly spaced points along the image width (i.e., two at the edges, one at midpoint). Pennation angle was measured along the VL muscle (i.e., at 20, 50, and 60% of thigh length), as the angle between the deep aponeurosis and the line of the fascicle. Moreover, VL fascicle length (FL) was estimated as:

(1) $$\begin{array}{r} FL\;=\;\frac{Thickness}{sin\;\theta } \end{array}$$

where θ is the fascicle pennation angle. Three pennation angle and FL measurements were performed on the two repeated images for each measurement site, and an average was taken to provide overall VL pennation angle and FL.

#### Countermovement-jump incremental test

After a standardized warm-up protocol (i.e., 5 min jogging, joint mobility exercises and five unloaded CMJ), participants completed an incremental CMJ test at five absolute loading conditions (i.e., 17, 30, 45, 60, and 75 kg) using a Smith Machine (Technogym, Gambettola, Italy). Two attempts were performed at each loading condition and resting time between repetitions and loads was set to 1 and 3 min, respectively. Instructions to keep constant downward pressure on the barbell and to jump as high as possible were given. Also, participants were instructed to perform a CMJ depth of ~90° knee flexion and to keep it constant across the different loading conditions. All jumps were performed on a force plate (Type 9260AA6, Kistler, Switzerland) which recorded vertical ground reaction force (GRF) data at 1 kHz, using an analog-to-digital converter (DAQ system 5691A1, Kistler). Signals were collected through the BioWare software (Kistler, Winterthur, Switzerland). For each jump, the system center of mass (COM) velocity was calculated from vertical GRF recordings (Linthorne, 2001). Specifically, net GRF was calculated by subtracting the system weight and then divided by the system mass (kg) to provide acceleration. Acceleration was numerically integrated to provide instantaneous COM velocity. Further analyses were performed on the concentric movement phase, defined from the onset of upward motion (i.e., instantaneous COM velocity > 0 m·s^−1^) to the take-off instant (i.e., GRF < 5 N). Only the repetition with the highest mean velocity and correct COM displacement was selected for further analysis. At each loading condition, mean power output was obtained and used for comparisons. Moreover, force and velocity were averaged across the concentric phase of each load and modeled by a least-squares linear regression model to calculate the FV profile variables. The intercepts of the FV relationship with the force and velocity axes were taken to calculate the maximal theoretical force (i.e., force value when velocity is 0; F_0_) and maximal theoretical velocity (i.e., velocity when force is 0; V_0_), respectively. The slope of the relationship (F_0_/V_0_) and maximal theoretical power (P_0_) (computed as P_0_ = (F_0_·V_0_)/4) were also analyzed.

#### Repeated countermovement jump test

Fifteen minutes after the completion of the FV incremental test, participants carried out a 15-repetition CMJ test at maximum intended velocity (Nindl et al., 2002). For each participant, the external load used during the test was individually selected as the load associated with barbell mean propulsive velocity of 1 m·s^−1^, interpolated from the load-velocity relationship performed on that day. All repetitions were performed continuously, and participants were verbally encouraged to jump as high as possible and to maintain CMJ depth constant during all repetitions. A linear velocity transducer (T-Force System; Ergotech, Murcia, Spain) providing barbell displacement instantaneous feedback was used to calculate mean propulsive power for each repetition. The mean propulsive power averaged across all 15 CMJ repetitions were measured and used for further comparisons (CMJ_15MP_).

All signal data analyses were performed using custom-written scripts computed with MATLAB (version R2015a; The Mathworks, Natick, Massachusetts, USA).

### Statistical analysis

Descriptive statistics are presented as mean ± standard deviation (SD). Normal distributions of the data were confirmed using a Shapiro-Wilk test. Between-day reliability of isometric torque, ultrasound and EMG parameters was assessed by calculating the intraclass correlation coefficient (ICC), coefficient of variation (CV) and corresponding 95% confidence intervals. Separate two-factor mixed ANOVAs were used to assess the effects of time (within-participant factor: pre *vs*. post) and training group (between-participants factor: IHRT *vs*. NT) on the FV-profile variables (i.e., *F*_0_, *V*_0_, *P*_0_, *Slope*), CMJ_15MP_, MVF, EMG_MVF_ and VL pennation angle and fascicle length. A three-factor mixed analysis of variance (ANOVA) was used to evaluate the effects of training group (between-participants factor: IHRT *vs*. NT), time (within-participant factor: pre *vs*. post) and loading condition (within-participant factor: 17, 30, 45, 60, and 75 kg) on power output. Separate three-factor mixed ANOVAs were employed to evaluate the effects of training group (between-participants factor: IHRT *vs*. NT), time (within-participant factor: pre *vs*. post) and contraction timing (i.e., F_50_, F_100_, F_150_, and EMG_0−50_, EMG_50−100_, and EMG_100−150_, in the isometric force and EMG variables, respectively) on explosive strength and EMG activation variables. Finally, a three-factor mixed ANOVA was also used to assess the effects of training group (between-participants factor: IHRT *vs*. NT), time (within-participant factor: pre *vs*. post) and muscle site (eight level within-participant factor: VL, RF, VI, and VM muscles at each corresponding thigh length: 20, 50, and 60%). Alpha was set at 0.05 for ANOVAs. Generalized Eta-Squared measures of effect size and thresholds (0.02 [small], 0.13 [medium], and 0.26 [large]) were calculated along with ANOVA effects (Bakeman, 2005).

In addition to the null-hypothesis statistical testing, standardized differences (i.e., Cohen's *d* effect sizes; thresholds: >0.2 [*small*], >0.6 [*moderate*], >1.2 [*large*], and *very large* [>2]; Hopkins et al., 2009) with 90% confidence intervals and qualitative probabilistic inferences indicating confidence (*possibly, likely, most likely, almost certainly*) and magnitude levels of the observed changes (*trivial, small, moderate, large, very large*) were calculated. All statistical analyses were performed using R software (version 3.3.2, R Core Team, 2017). Packages “*ez*” (Lawrence, 2016) and “*mbir”* (Peterson, 2017) were employed to perform ANOVA and magnitude-based inferences, respectively.

## Results

### Reliability of isometric and muscle architecture measurements

Reliability data on the isometric strength, voluntary activation and muscle architecture variables is shown in Table 2.

**Table 2:** Reliability outcomes of isometric strength (N), electromyography (EMG_0−50_, EMG_50−100_, and EMG_50−100_ [% MVC_EMG_]; EMG_MVC_ [mV]) and muscle architecture (RF, VI, VL, and VM muscles thickness at 20, 50, or 60% of thigh length [mm]; pennation angle [°] and fascicle length [mm]) variables measured from the two repeated measurements prior to the training intervention.

	**Test 1 Mean ± SD**	**Test 2 Mean ± SD**	**CV % (95% CI)**	**ICC (95% CI)**
**ISOMETRIC STRENGTH**				
MVF	890.0 ± 187.3	883.2 ± 181.4	3.76 (2.3, 5.22)	0.97 (0.93, 0.99)
F_50_	151.4 ± 70.3	176.0 ± 52.6	24.71 (14.21, 35.21)	0.68 (0.2, 0.87)
F_100_	459.4 ± 145.8	481.6 ± 101.1	11.39 (5.12, 17.66)	0.86 (0.66, 0.94)
F_150_	621.1 ± 175.3	631.1 ± 134.0	8.69 (4.01, 13.36)	0.90 (0.75, 0.96)
**ELECTROMYOGRAPHY**				
EMG_0−50_	60.3 ± 26.6	68.2 ± 18.7	23.5 (12.62, 34.38)	0.52 (-0.18, 0.81)
EMG_50−100_	87.6 ± 25.6	95.1 ± 21.4	17.3 (11.17, 23.43)	0.45 (-0.35, 0.78)
EMG_100−150_	77.8 ± 18.8	83.1 ± 18.6	14.3 (8.86, 19.74)	0.56 (-0.08, 0.82)
EMG_MVC_	0.23 ± 0.10	0.25 ± 0.12	9.44 (6.03, 12.86)	0.97 (0.93, 0.99)
**MUSCLE ARCHITECTURE**				
RF_50_	23.8 ± 2.9	23.6 ± 2.9	2.3 (1.13, 3.48)	0.96 (0.9, 0.98)
RF_60_	25.3 ± 2.5	24.7 ± 2.8	3.27 (2.13, 4.41)	0.93 (0.82, 0.97)
VI_50_	21.5 ± 4.1	21.5 ± 4.3	3.15 (2.02, 4.28)	0.98 (0.95, 0.99)
VI_60_	22.2 ± 4.5	22.3 ± 5.1	4.02 (2.1, 5.93)	0.96 (0.91, 0.98)
VL_20_	17.4 ± 5.7	17.9 ± 4.7	8.81 (3.59, 14.02)	0.93 (0.83, 0.97)
VL_50_	25.7 ± 2.6	25.8 ± 2.9	2.72 (1.75, 3.68)	0.95 (0.87, 0.98)
VL_60_	27.7 ± 2.9	26.9 ± 2.8	2.92 (1.64, 4.2)	0.93 (0.84, 0.97)
VM_20_	20.7 ± 4.7	20.9 ± 5.1	6.36 (3.65, 9.07)	0.94 (0.85, 0.98)
VL P. Angle	11.7 ± 1.7	12.5 ± 1.4	6.99 (4.65, 9.33)	0.84 (0.61, 0.94)
VL F. Length	124.2 ± 22.3	113.3 ± 18.9	8.33 (5.36, 11.31)	0.83 (0.61, 0.93)

### Muscle architecture

**Figure 3:**
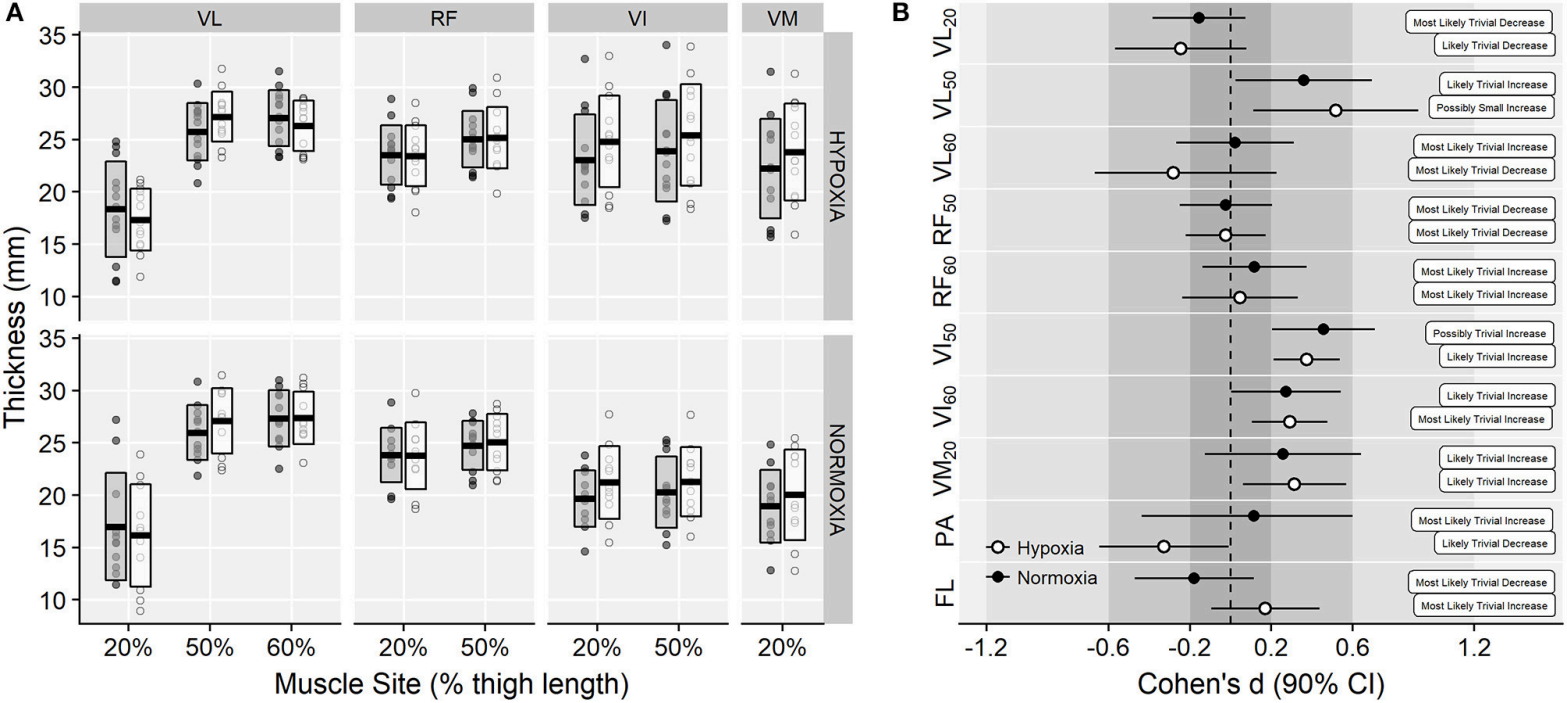
**(A)** Vastus lateralis (VL), rectus femoris (RF), vastus intermedius (VI), and vastus medialis (VM) thickness raw values at each corresponding percentage of thigh length before (gray-filled boxes) and after (white boxes) training. Horizontal thick lines within bars are mean and upper and lower bars edges define standard deviation. Individual observations are shown as gray or white circles, respectively. **(B)** Cohen's d standardized mean differences with 90% confidence intervals and magnitude-based inferences for the muscle thickness (subscripts indicate the respective location of measurement relative to thigh length), pennation angle (PA), and fascicle length (FL) variables.

Muscle thickness values for each corresponding training group are shown in Figure 3A. ANOVA showed a main effect of muscle site (*F* = 38.00, *p* < 0.001, $\eta_{\text{G}}^{2}$ = 0.43), and a time × muscle site interaction effect (*F* = 10.85, *p* < 0.001, $\eta_{\text{G}}^{2}$ = 0.02) on muscle thickness, due to *small* increments in VL_50_, VI and VM muscles (Figure 3B). No main effect of time (*F* = 3.36, *p* = 0.080, $\eta_{\text{G}}^{2}$ < 0.01), training group × time (*F* = 0.01, *p* = 0.939, $\eta_{\text{G}}^{2}$ < 0.01) or training group × time × muscle site interaction effects were observed on muscle thickness (*F* = 0.56, *p* = 0.722, $\eta_{\text{G}}^{2}$ < 0.01).

No main effects of time (12.1 ± 1.4 *vs*. 11.9 ± 1.3°, pre to post-training respectively; *F* = 0.29, *p* = 0.596, $\eta_{\text{G}}^{2}$ < 0.01) or training group (*F* = 0.03, *p* = 0.871, $\eta_{\text{G}}^{2}$ < 0.01) were observed on VL pennation angle. A small training group × time effect was observed (*F* = 3.06, *p* = 0.094, $\eta_{\text{G}}^{2}$ = 0.02) due to *small* decrements in the IHRT group (12.3 ± 1.3 *vs*. 11.8 ± 1.3° and 11.8 ± 1.6 *vs*. 12.1 ± 1.4°, in the IHRT and NT groups, respectively; Figure 3). Similarly, no main effects of time (*F* = 0.07, *p* = 0.790, $\eta_{\text{G}}^{2}$ < 0.01) and a trivial training group × time interaction effect (115.2 ± 16.8 *vs*. 118.2 ± 16.2 mm and 120.5 ± 22.2 *vs*. 116.3 ± 21.4 mm, in the IHRT and NT groups, respectively; *F* = 2.57, *p* = 0.123, $\eta_{\text{G}}^{2}$ < 0.01) were observed on fascicle length (Figure 3B).

### Isometric force

**Figure 4:**
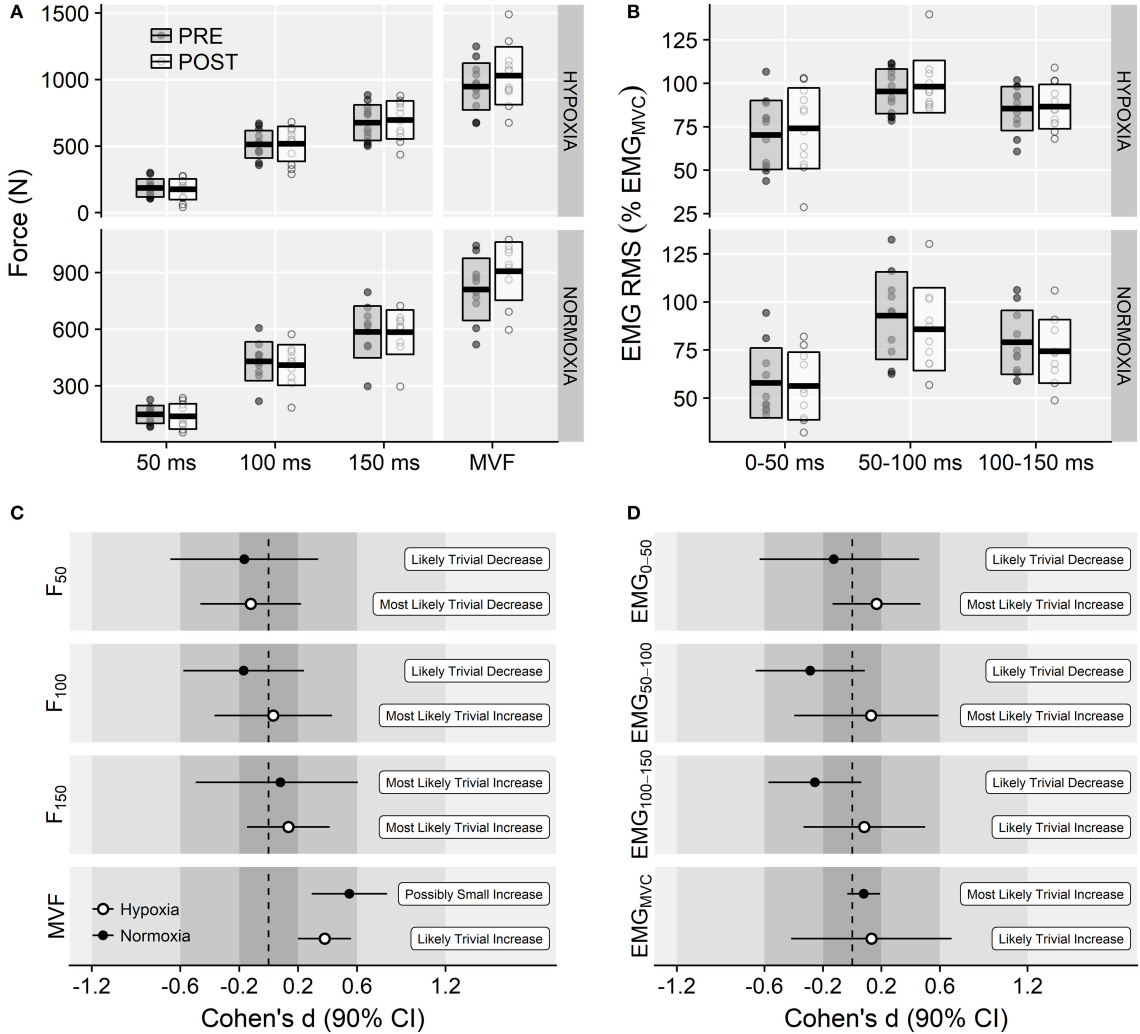
**(A)** Isometric explosive and maximal force values before and after the training intervention at each corresponding environmental condition. **(B)** Voluntary activation levels (EMG RMS normalized to EMG_MVC_) before and after the training intervention. In top panels, horizontal thick lines within bars are mean and upper and lower bars edges define standard deviation. Individual observations are shown as gray or white circles, respectively. Panels **(C,D)** display magnitude-based inferences for the isometric strength and EMG activation variables.

There was a main effect of time on MVF (*F* = 29.96, *p* < 0.001, $\eta_{\text{G}}^{2}$ = 0.06) due to increments in both experimental groups. No main effect of training group (*F* = 2.89, *p* = 0.104, $\eta_{\text{G}}^{2}$ = 0.12) or time × training group interaction (*F* = 0.22, *p* = 0.645, $\eta_{\text{G}}^{2}$ < 0.01) were observed on MVF (Figure 4A). Likewise, no main effects of time (*F* = 0.04, *p* = 0.853, $\eta_{\text{G}}^{2}$ < 0.01) or time × training group interaction effects (*F* = 0.25, *p* = 0.620, $\eta_{\text{G}}^{2}$ < 0.01) were observed on the explosive isometric strength (Figure 4A). Magnitude-based inferences are shown in Figure 4C.

### Electromyography (EMG)

No main effects of time (*F* = 0.64, *p* = 0.432, $\eta_{\text{G}}^{2}$ < 0.01) or training group (*F* = 0.17, *p* = 0.686, $\eta_{\text{G}}^{2}$ < 0.01) were found on EMG_MVF_. Likewise, there was no training group × time interaction effect (0.22 ± 0.07 *vs*. 0.23 ± 0.08 mV in the IHRT group and 0.24 ± 0.15 *vs*. 0.25 ± 0.13 mV in the NT group, pre to post-training, respectively; *F* = 0.00, *p* = 0.952, $\eta_{\text{G}}^{2}$ < 0.01). ANOVA showed no main effects of time (*F* = 0.04, *p* = 0.853, $\eta_{\text{G}}^{2}$ < 0.01) or time × training group interaction effect on the EMG activation during the explosive contractions (*F* = 0.25, *p* = 0.620, $\eta_{\text{G}}^{2}$ < 0.01; Figures 4B,D).

### Force-velocity test

**Figure 5:**
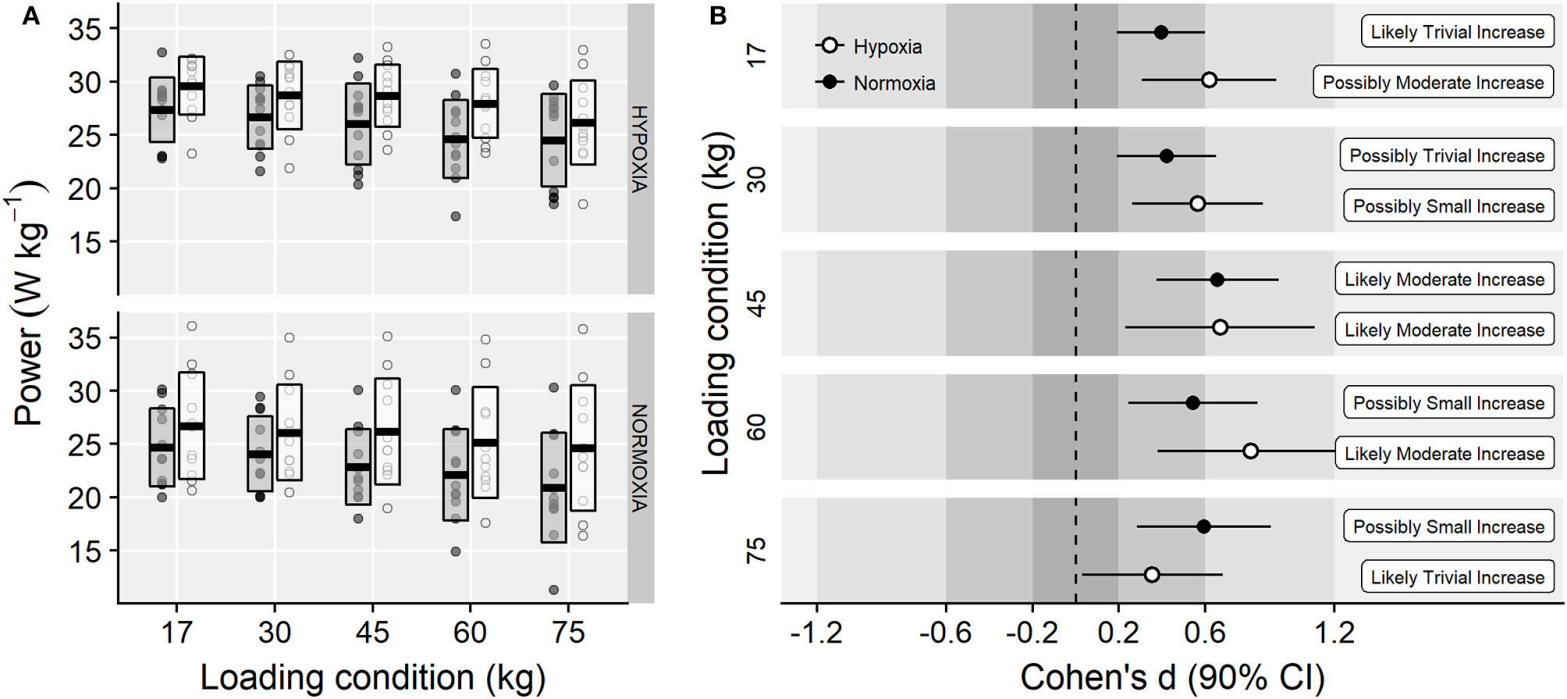
**(A)** Power output values at each loading condition before (gray-filled boxes) and following (white boxes) training. Horizontal thick lines within bars are mean and upper and lower bars edges define standard deviation. Individual observations are shown as gray or white circles, respectively. **(B)** Cohen's d standardized mean differences and 90% confidence intervals for each corresponding variable.

Main effects of time (*F* = 30.91, *p* < 0.001, $\eta_{\text{G}}^{2}$ = 0.10) and loading condition (*F* = 34.18, *p* < 0.001, $\eta_{\text{G}}^{2}$ = 0.07) were observed on power output. However, no meaningful training group × time (*F* = 0.22, *p* = 0.647, $\eta_{\text{G}}^{2}$ < 0.01), time × load (*F* = 1.97, *p* = 0.132, $\eta_{\text{G}}^{2}$ < 0.01), training group × load (*F* = 0.08, *p* = 0.932, $\eta_{\text{G}}^{2}$ < 0.01) or training group × load × time (*F* = 1.89, *p* = 0.145, $\eta_{\text{G}}^{2}$ < 0.01) interaction effects were observed (Figure 5).

**Figure 6:**
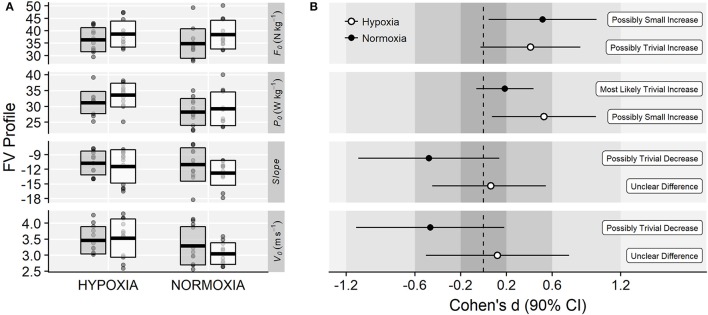
**(A)** Raw values of the FV profile related variables before (gray-filled boxes) and after (white boxes) training. *Slope* units are N·s·m^−1^·kg^−1^. Horizontal thick lines within bars are mean and upper and lower bars edges define standard deviation. Individual observations are shown as gray or white circles, respectively. **(B)** Cohen's d standardized mean differences and 90% confidence intervals for each corresponding variable.

Regarding the FV profile parameters, significant main effects of time were observed on *F*_0_ (35.6 ± 5.4 *vs*. 38.5 ± 5.4 N·kg^−1^; *F* = 6.55, *p* = 0.018, $\eta_{\text{G}}^{2}$ = 0.07) and *P*_0_ (29.8 ± 4.1 *vs*. 31.5 ± 5.0 W·kg^−1^; *F* = 6.45, *p* = 0.019, $\eta_{\text{G}}^{2}$ = 0.04). There was a main effect of training group on *P*_0_ (*F* = 4.94, *p* = 0.037, $\eta_{\text{G}}^{2}$ = 0.17) due to greater values in the IHRT compared to the NT group (32.4 ± 3.2 *vs*. 28.7 ± 4.6 W·kg^−1^). No main effects of time on *V*_0_ (3.38 ± 0.51 *vs*. 3.30 ± 0.54 m·s^−1^; *F* = 0.43, *p* = 0.521, $\eta_{\text{G}}^{2}$ < 0.01), *Slope* (−10.9 ± 2.9 *vs*. −12.1 ± 3.0 N·s·m^−1^·kg^−1^; *F* = 2.19, *p* = 0.153, $\eta_{\text{G}}^{2}$ = 0.04) or time × training group interactions were observed on any of the FV profile variables (*F*_0_: *F* = 0.33, *p* = 0.569, $\eta_{\text{G}}^{2}$ < 0.01; *V*_0_: *F* = 1.33, *p* = 0.261, $\eta_{\text{G}}^{2}$ = 0.03; *P*_0_: *F* = 1.04, *p* = 0.320, $\eta_{\text{G}}^{2}$ < 0.01; *Slope*: *F* = 0.39, *p* = 0.541, $\eta_{\text{G}}^{2}$ < 0.01; Figure 6A). Magnitude-based inferences are displayed in Figure 6B.

### Repeated CMJ test (CMJ_15MP_)

**Figure 7:**
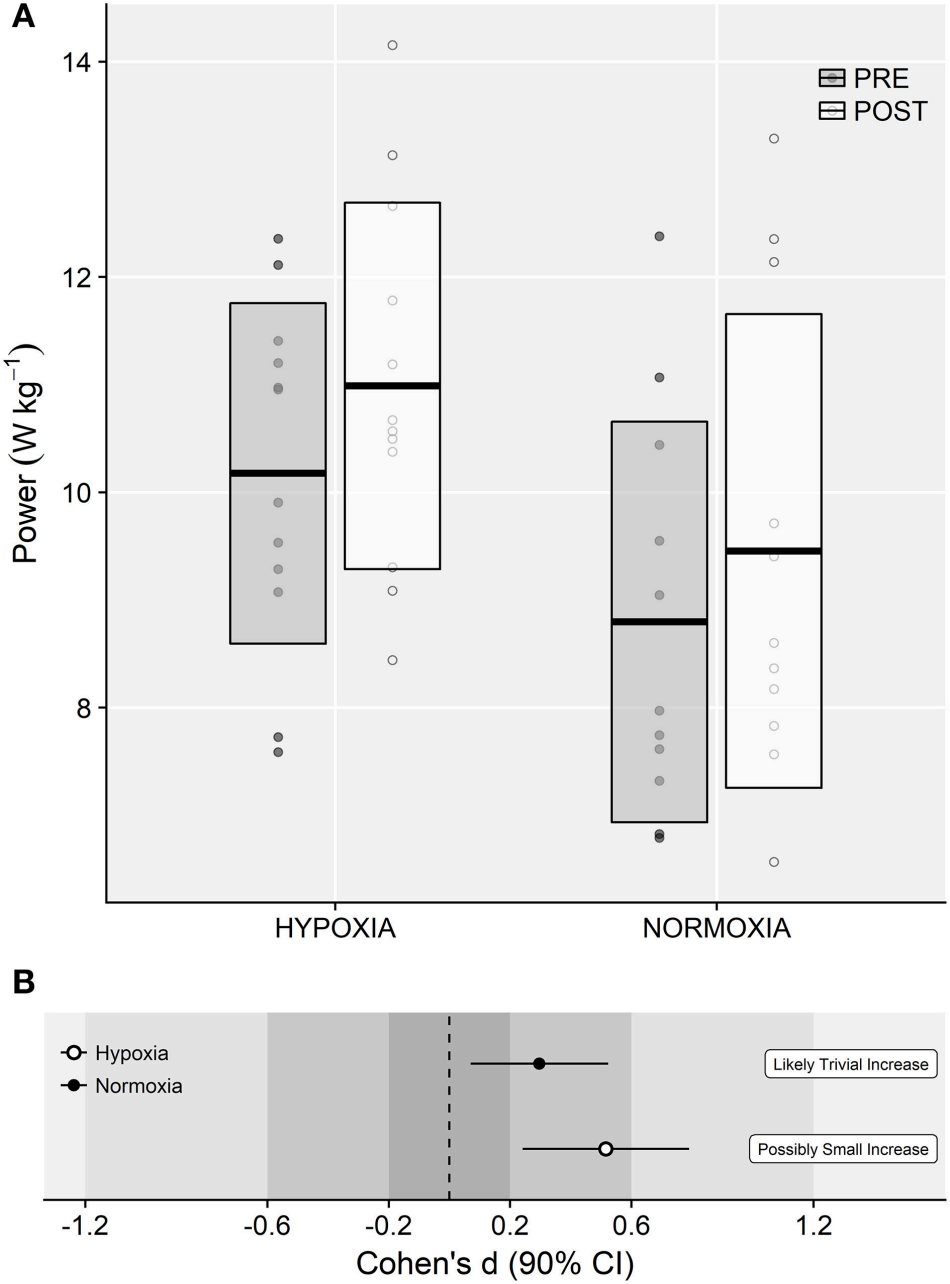
**(A)** Mean power values obtained during the repeated CMJ test (CMJ_15MP_). Horizontal thick lines within bars are mean and upper and lower bars edges define standard deviation. Individual observations are shown as gray or white circles, respectively. **(B)** Cohen's *d* standardized mean differences and 90% confidence intervals for each corresponding variable.

ANOVA showed a main effect of time on CMJ_15MP_ (9.5 ± 1.8 *vs*. 10.3 ± 2.1 W·kg^−1^; *F* = 16.15, *p* < 0.001, $\eta_{\text{G}}^{2}$ = 0.04). A small main effect of training group was observed (*F* = 3.81, *p* = 0.064, $\eta_{\text{G}}^{2}$ = 0.15), although no meaningful time × training group interaction effects were observed (*F* = 0.18, *p* = 0.679, $\eta_{\text{G}}^{2}$ < 0.01; Figures 7A,B).

## Discussion

The present study examined the effects of an 8-week resistance training intervention, under normoxic or moderate altitude environmental conditions (i.e., intermittent exposure), on the functional (i.e., maximal and explosive strength performance), voluntary EMG activation and morphological adaptations of the quadriceps muscles. The main results, specific to the training strategy employed, showed similar functional and morphological training muscle responses in both environmental conditions. Despite no meaningful between-group statistical differences, opposite trends in pennation and fascicle length changes were depicted.

Compared to NT, the IHRT intervention exhibited similar maximal strength responses assessed either from the FV-relationship (i.e., *F*_0_) or using a single-leg knee extension isometric MVC. These results contribute to the conflicting IHRT research available showing significant (Inness et al., 2016; Yan et al., 2016) and non-meaningful changes in maximal strength compared to training under normoxic conditions (Ho et al., 2014; Kurobe et al., 2015). The wide variety of training protocols and strength assessment procedures used in previous research [i.e., dynamic: direct (Inness et al., 2016) and indirect (Nishimura et al., 2010) estimations of 1RM; static: bilateral (Yan et al., 2016) and unilateral (Manimmanakorn et al., 2013a) isometric MVF] make direct comparisons difficult. Strength training responses are known to be load and task-specific (Cormie et al., 2010a), which may partially explain the conflicting evidence often reported in the current IHRT literature. The five-load FV-profile assessment procedure used in this investigation shows that training adaptations were maximized in the loading conditions close to the training load used (i.e., greatest changes in power output in 60 kg; Figure 5), although no meaningful differences between loading conditions were observed. Indeed, while “*small*” to “*moderate*” changes in F_0_ and MVF occurred in both groups, unclear changes in V_0_ and unchanged isometric explosive performance were observed. Consequently, “*small*” changes in the overall FV-performance (i.e., P_0_) were observed only in the IHRT group, although group differences with NT were trivial (see Figure 6). It should be noted that these responses are consistent with the training load adjustment approach used. The fact that training velocity was kept at 1 m·s^−1^ and thus the training load increased throughout the training period likely explains why changes in maximal but no explosive strength parameters (i.e., F_50_, F_100_, F_150_, V_0_) were observed in both groups.

Neuromuscular and muscle morphological changes are known to underpin changes in muscle strength following resistance training (Folland and Williams, 2007). The use of IHRT to maximize muscle growth remains one of the potential training applications (Nishimura et al., 2010; Deldicque and Francaux, 2013; Manimmanakorn et al., 2013a) even in the absence of muscle functional adaptations (Kurobe et al., 2015). In the present study, however, no differences between training conditions were observed on the muscle thickness changes. Both groups showed region-specific morphological changes, with “*small*” changes in the thickness of VL and VI muscles (see Figure 3). The small magnitude of the observed muscle thickness changes after both IHRT and NT is likely explained by the high-intensity, power-oriented (Blazevich et al., 2003) and short-term nature of the training intervention employed (Blazevich et al., 2007). Despite unclear differences between groups, a tendency toward smaller VL pennation angles and greater fascicle lengths in the IHRT group was observed, which has been previously linked with velocity-specific muscle architecture responses (Blazevich et al., 2003). Variations in the amount of skeletal muscle contractile tissue are known to underpin changes in muscle architecture (Blazevich and Sharp, 2005; Narici et al., 2016). However, while training-specific changes in muscle architecture could be expected (Blazevich et al., 2003), neither the training period length nor the sets and repetitions schemes employed in the current investigation were designed to specifically target skeletal muscle growth (Schoenfeld et al., 2017). Instead, a combination of neuromuscular and structural changes of the muscle-tendon unit is known to determine muscle power production (Cormie et al., 2010a), which are likely to account for the improvements in strength performance observed here. Notwithstanding, no significant changes in voluntary activation were observed during isometric maximal and explosive strength assessments. Task-specific adaptations (Buckthorpe et al., 2015), as well as the high between-day variability observed in the EMG variables, may explain the lack of neural effects observed in muscle activation.

It should be considered that the current experimental design did not comprise a mid-intervention assessment, which would have allowed to examine potential differences in the time course of training-induced responses. While neural adaptations are thought to be responsible for the early responses to resistance training (Moritani and deVries, 1979; Folland and Williams, 2007), other biomechanical factors not comprised in the current investigation, should have been also considered to reach a comprehensive evaluation of the mechanisms determining muscle performance. Task-specific neuromuscular adaptations (i.e., changes in muscle coordination) and changes in the mechanical properties of the muscle-tendon unit may have occurred and should be addressed in future investigations.

Collectively, the results of the present investigation display similar responses in terms of muscle strength and muscle architectural adaptations following an 8-week resistance training intervention. Nonetheless, these and previous findings suggest that, contrary to earlier adverse associations between altitude and resistance-training muscle responses (Narici and Kayser, 1995), similar anatomical and functional muscle strength responses can be achieved in both environmental conditions. These results may be of relevance for athletes and coaches planning their altitude training strategies.

## Author contributions

AM-A, PP, BD, BD, and BF contributed to the conception and design of the study. All authors, AM-A, PP, AG-R, AP-C, JA-C, BD, and BF participated in data base collection. AM-A and BF organized the database and performed the statistical analysis. AM-A wrote the first draft of the manuscript. BF supervised the study. All authors contributed to manuscript revision, read and approved the submitted version.

### Conflict of interest statement

The authors declare that the research was conducted in the absence of any commercial or financial relationships that could be construed as a potential conflict of interest.
